# Numerical Study of the Coupling of Sub-Terahertz Radiation to n-Channel Strained-Silicon MODFETs

**DOI:** 10.3390/s21030688

**Published:** 2021-01-20

**Authors:** Jaime Calvo-Gallego, Juan A. Delgado-Notario, Jesús E. Velázquez-Pérez, Miguel Ferrando-Bataller, Kristel Fobelets, Abdelaziz El Moussaouy, Yahya M. Meziani

**Affiliations:** 1 NanoLab, Universidad de Salamanca, Plaza de la Merced, Edificio Trilingüe, 37008 Salamanca, Spain; jaime.calvo@usal.es (J.C.-G.); juanandn@usal.es (J.A.D.-N.); js@usal.es (J.E.V.-P.); 2 Departament of Communications, Telecommunication Engineering School, Universitat Politècnica de València, 46022 Valencia, Spain; mferrand@dcom.upv.es; 3 Department of Electrical and Electronic Engineering, Imperial College London, Exhibition Road, London SW7 2BT, UK; k.fobelets@imperial.ac.uk; 4 Department of Physics, Faculty of Sciences, Mohammed I University, Oujda 60000, Morocco; azize10@yahoo.fr

**Keywords:** terahertz, SiGe, silicon, strained-Si, MODFET, electromagnetic simulation

## Abstract

This paper reports on a study of the response of a T-gate strained-Si MODFETs (modulation-doped field-effect transistor) under continuous-wave sub-THz excitation. The sub-THz response was measured using a two-tones solid-state source at 0.15 and 0.30 THz. The device response in the photovoltaic mode was non-resonant, in agreement with the Dyakonov and Shur model for plasma waves detectors. The maximum of the photoresponse was clearly higher under THz illumination at 0.15 THz than at 0.3 THz. A numerical study was conducted using three-dimensional (3D) electromagnetic simulations to delve into the coupling of THz radiation to the channel of the transistor. 3D simulations solving the Maxwell equations using a time-domain solver were performed. Simulations considering the full transistor structure, but without taking into account the bonding wires used to contact the transistor pads in experiments, showed an irrelevant role of the gate length in the coupling of the radiation to the device channel. Simulations, in contradiction with measurements, pointed to a better response at 0.3 THz than under 0.15 THz excitation in terms of the normalized electric field inside the channel. When including four 0.25 mm long bonding wires connected to the contact pads on the transistor, the normalized internal electric field induced along the transistor channel by the 0.15 THz beam was increased in 25 dB, revealing, therefore, the important role played by the bonding wires at this frequency. As a result, the more intense response of the transistor at 0.15 THz than at 0.3 THz experimentally found, must be attributed to the bonding wires.

## 1. Introduction

Electromagnetic radiation in the terahertz (THz) spectral range has the potential to offer vast improvements on the scope and performance of many devices and systems, from avionics to medical instruments. The THz frequency bands are usually considered to sit between the millimeter wave band at ~0.3 mm or 0.1 THz and the far infrared band at ~30μm or 10 THz. It is often known as the “THz gap” because of the historic relative inability to research and apply it in comparison to the radio and optical wave bands on either side. 

Among the inherent properties that make THz radiation attractive for applications the following can be listed: ability to penetrate non-conducting materials, safer and more accurate method of medical diagnostic imaging than X-rays, capability to detect objects through clothing or plastic bags, capability of non-destructive measurement of multi-layered materials, stability in high-altitude communication applications (particularly satellite applications), non-destructive and non-ionizing, etc. So far, many different applications in the THz range have been investigated and demonstrated [1] in different fields as: spectroscopy [2], astronomy [3], communications [4], screening and security [5], metrology [6], etc.

In a recent roadmap of the development of THz technology, [7], semiconductor-based THz detectors have been identified as a key component in future THz systems. Throughout the past years, the progress in new semiconductor materials and devices has fueled the research of room-temperature terahertz detectors [8]. 

Currently, plasma wave terahertz electronics, based on a pioneering work of Dyakonov and Shur [9,10] that proposed the use of the nonlinear properties of the two-dimensional (2D) electron plasma in the channel of a FET (field-effect transistor) to detect signals in bands beyond the cut-off frequency of the transistor, is one of the most promising ways to achieve direct detection of THz beams using solid-state devices at room temperature. Direct detection of sub-THz radiation has already been demonstrated at room temperature using different types of FETs based on silicon such as conventional silicon MOSFETs [11] and strained-Si MODFETs [12]. But, across the last twenty years many efforts have been devoted to the development of semiconductor-based THz direct detectors: resonant detection by GaAs/AlGaAs FETs was first reported by Knap et al. [13] at 8 K that also reported on non-resonant detection at room temperature [14]. In 2004, room temperature, non-resonant detection by silicon field-effect transistors was demonstrated for the first time [15]. THz imaging based on CMOS technology has been reported by different groups [16,17]. In 2009, an array built using 0.25 mm CMOS technology with responsivity of 80 kV/W and NEP of 300 pW/pHz that was used for imaging at 0.65 THz was reported [18]. 

The present paper reports on the use of Schottky-gated FETs based on the SiGe/Si/SiGe double-heterojunction as sub-THz plasma-waves detectors. According to the Shur and Dyakonov model, [10], a FET’s channel acts as a resonator for plasma waves and under electromagnetic radiation a DC drain-to-source voltage will be induced for specific boundary conditions: the source of the transistor is grounded and the drain is in open-circuit (photovoltaic mode). This DC drain to source voltage transducing the incoming THz radiation is usually measured using a lock-in technique. In the present work a T-gate Si/Si_0.7_Ge_0.3_ MODFET was used to detect a continuous wave (cw) THz radiation in the photovoltaic mode. 

The paper is organized as follows: Section 2 presents the Si/SiGe FET that is characterized at 0.15 and 0.30 THz along with the set up used. Section 3 presents the experimental results of the sub-THz characterization of the transistor along with a 3D FDTD (finite-difference time-domain) numerical study of the coupling of the THz beam to the transistor channel. The numerical study focuses on the role played by the metallic contacts and the bonding wires in the coupling of the radiation to the transistor channel. 

## 2. Materials and Methods

This section presents a short description of the strained-Si MODFETs (modulation-doped FETs) used in this work as sub-THz detectors along with the description of the experimental setup used the in sub-THz characterization of the transistor.

### 2.1. n-Type MODFETs Based on Si/SiGe Heterojunctions

**Figure 1:**
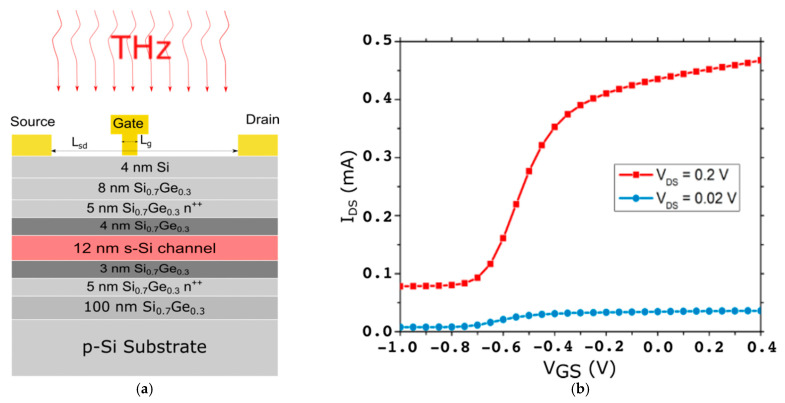
(**a**) Cross section of the Si/SiGe n-channel modulation-doped field-effect transistor (MODFETs) showing the vertical layout of the transistor with a schematic of the contacts. The strained-Si layer is highlighted in carnation color while the two supply layers are indicated with n^++^; (**b**) measured transfer characteristics of the Si/SiGe n-channel MODFETs at two different values of the drain-to- source (V_DS_) biases: 20 and 200 mV.

Modulation-doped FETs (MODFETs) use a single or a double heterojunction between two materials with different bandgaps. A thin layer of the semiconductor with the larger bandgap of the heterojunction material system is degenerately doped to supply carriers (electrons in a n-channel FET) to a non-intentionally doped layer made of the semiconductor with the lower bandgap. This last layer is used to define the channel of the transistor. The electron transfer to the channel layer is supported by the conduction (ΔE_C_) and (ΔE_V_) valence band discontinuities of the heterojunction that also contribute to the confinement of majority carries in the channel. The MODFET used as THz detector in this work (Figure 1) is based on the Si/SiGe material system that, for a Ge mole fraction of 0.3, leads to a value of ΔE_C_ in the heterojunction Si/Si_0.70_Ge_0.30_ of about 180 meV ensuring an excellent electron confinement in the strained-Si quantum well. In this transistor a double heterojunction SiGe/Si/SiGe is used to, on the one hand, establish a tetragonal (biaxial tensile) strain on the internal silicon layer (i.e., the transistor channel) and, on the other hand, create two supply layers in the two unstrained SiGe layers around the channel. The combination of the modulation doping, the strain of the channel layer, the dual-supply definition, and the asymmetrical placement of the gate between source and drain (Figure 1b) ensure an excellent performance of the strained-Si MODFET as a sub-THz detector [19,20,21] that will allow it to compete with detectors based on other technologies. These technologies include, among others, THz detectors based on mainstream CMOS [22] that have achieved responsivities close to 350 V/W at 0.6 THz, plasmonic detectors based on InAlAs/InGaAs/InP high electron mobility transistors (HEMTs) with an asymmetric dual-grating-gate structure exhibiting a responsivity of 22.7 kV/W at 200 GHz in the photovoltaic mode [23,24] at room temperature and forthcoming technologies based on two-dimensional materials [25].

Figure 1a presents the vertical layout of the MODFETs whose sub-THz response is studied in this work along with the schematic of the overhead front THz illumination. Further details related to this vertical layout and device performance are given in [21]. The main geometrical parameters of the transistor were: the total drain-to-source distance (*L_sd_*) was 2 μm, the gate width (*W_g_*) was 60 μm, the separation between the right edge of the source contact and the left edge of the gate (*L_gs_*) was 1 μm, and the gate length (*L_g_*) was *L_g_* = 250 nm. Figure 1b gives the measured transfer characteristics of the transistor at two drain-to-source biases (V_DS_ = 0.2 and 0.02 V).

### 2.2. Terahertz Characterization

**Figure 2:**
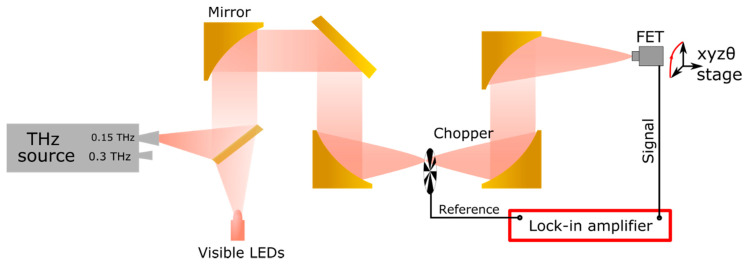
Schematics of the setup used in the sub-THz measurements: a solid-state source (at the left side of the figure) generates electromagnetic radiation at 0.15 and 0.3 THz that is detected by the strained-Si MODFET mounted on a XYZθ stage (at the right side of the figure). The chopper and the lock-in amplifier used to detect the signal generated by the transistor under THz excitation are also shown.

The outline of the experimental setup used in the characterization of the strained-Si MODFET as a sub-THz detector is shown in Figure 2. The system is described in more detail in [14,15,16]. A solid-state harmonic generator with output power levels of 6 mW at 0.3 THz and 3 mW at 0.15 THz respectively was used as the exciting source. The THz beam was modulated by a mechanical chopper at 298 Hz. The beam was subsequently collimated and focused by off-axis parabolic mirrors. The red LED showed in the figure is used for alignment purposes before measurements. All THz measurements were carried out at room temperature.

**Figure 3:**
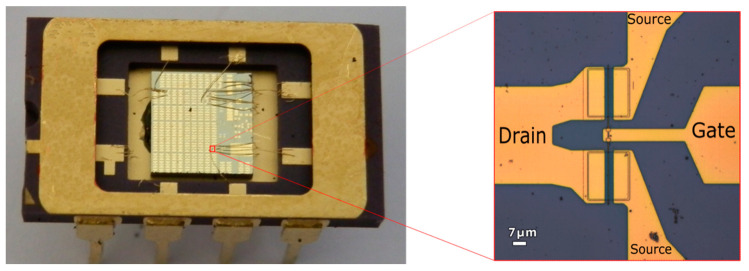
(**Left**): The chip containing the strained-Si MODFETs used in this work mounted on a dual in line package (DIP8) and gold wire-bounded to it. (**Right**): Magnified view of a transistor showing the T-gate.

The transistor under study was attached to the frame of a dual in-line package (DIP14/8) and the wire-bounded using gold wires as shown in Figure 3 and described in [15,16]. As no antennas coupled to the MODFET were used to enhance the THz detection, the radiation should be coupled to the device through the bonding wires and/or the metallic contacts on the chip (contact pads). The photo-induced drain-to-source voltage, Δ*U*, was measured using the lock-in technique. The lock-in amplifier used in the set up was a Stanford Research SR830. 

## 3. Results and Discussion

This section presents and discusses the results of a numerical study of the sub-THz response of strained-Si MODFETs under front illumination and compares them to the experimental ones. 

Two tones (0.15 and 0.3 THz) were used to characterize the sub-THz response of the MODFET device. The measurements were conducted in open air at room temperature using a solid-state cw terahertz source. It is based on a dielectric resonant oscillator (DRO) at 12.5 GHz and different stages of multipliers to reach 0.15 THz with a power of 3 mW and 0.3 THz with a power of 6 mW [14]. Prior to the characterization of the transistor, a highly sensitive calibrated pyroelectric detector was used to measure the power emitted by the source at its output. The incoming THz radiation was modulated by a mechanical chopper at 298 Hz, collimated and focused by off-axis parabolic mirrors. A red LED in combination with an indium tin oxide (ITO) mirror was used for the alignment of the THz beam. Both the LED and ambient light were switched off during measurements.

**Figure 4:**
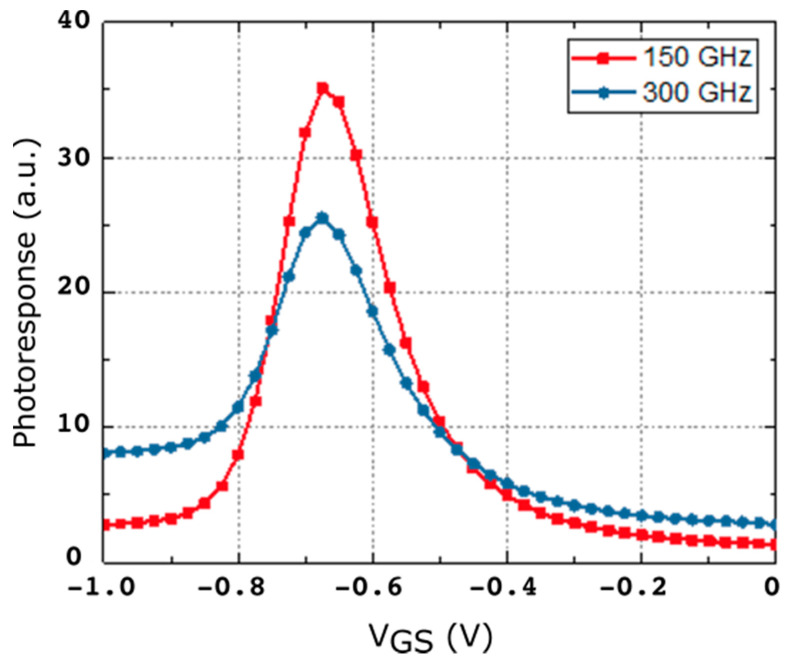
Photoresponse signal vs. gate voltage under front excitation of 0.15 THz (red symbols) and 0.3 THz (blue symbols).

Figure 4 shows the measured photoresponse at 0.15 THz and 0.3 THz. The maximum of the photoresponse signal was found, for both frequencies, when biasing the gate around the threshold voltage (~−0.67 V) of the transistor. This behavior has been reported earlier [15], and explained as non-resonant (broadband) detection. Bearing in mind that the output power of the source at 0.15 THz is one half of the one at 0.3 THz, the maximum photo-response of the strained-Si detector is almost three times more intense under excitation at 0.15 THz than at 0.3 THz (Figure 4). This fact was attributed to a more efficient coupling of the incoming THz radiation to the transistor channel at 0.15 THz [26]. 

**Figure 5:**
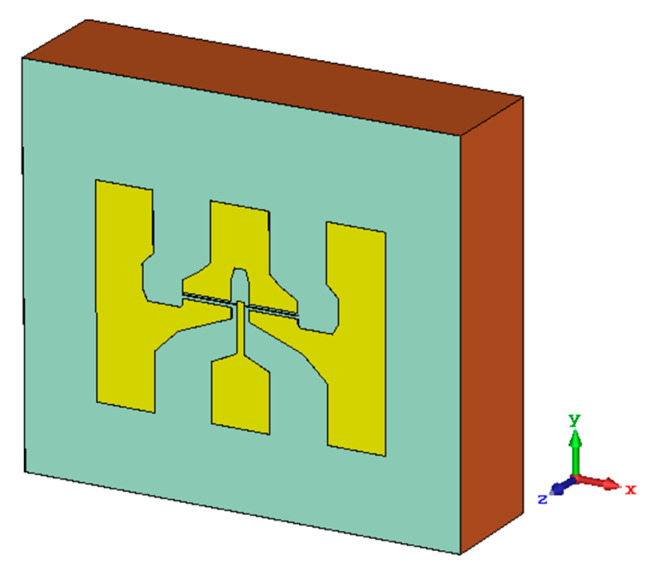
3D view of the structure used in the simulation, the three spatial axis are shown.

This work presents a numerical study using three-dimensional (3D) electromagnetic simulations to delve into the coupling of THz radiation to the channel of the strained-silicon MODFET. A model of the transistor was built in the electromagnetic solver software package CST^TM^ Microwave Studio. 3D simulations solving the Maxwell equations using the time domain solver were performed assuming that a plane wave with a frequency of 0.15 or 0.3 THz propagates along the z axis with normal incidence (front illumination) on the top side of the transistor (plane *x*-*y* in simulations) [15]. Figure 5 presents the volume simulated along with the three spatial axis. The origin of the z-axis is placed at the top surface of the simulated structure. 

The vertical layout of the device defined in the model is the one presented in Figure 1a. The thickness of the gold layer used to fabricate the top electrodes and contact pads was 300 nm. A top passivation layer of SiO_2_ with a thickness of 4 mm was added on top of the metallization layer. In simulations it is assumed that the simulated structure (Figure 5) was surrounded by air. Four values of *L_g_* were studied (0.50, 0.25, 0.15, and 0.10 μm) in order to investigate if the transistor gate itself has a significant impact on the coupling of THz radiation to the channel. 

The electric field magnitude of the incoming wave was kept equal to 1 V/m in all the simulations. Two cases were considered in the simulations: a first case in which the incoming electric field was considered as oriented along the x axis (perpendicular to transistor channel in the x-y plane), and a second one, in which the incoming field was oriented along the y axis (parallel to the transistor channel in the x-y plane); any orientation of the incoming radiation electric field can be expressed as a linear combination of those two orientations. It should however be noted that, as the response of the detector is nonlinear it cannot be obtained as a simple superposition of the individual responses of the detector to two waves orthogonally polarized.

Table 1 and Table 2 give the maximum values of the normalized internal electric field (*E_r_*) induced by the impinging THz beam along the transistor channel along the x and y axis, respectively. *E_r_* is given in dB as:

(1) $$E_{r}\left(dB\right)=20{log}_{10}\left(\frac{E}{\frac{1V}{m}}\right)$$

where the magnitude of the local electric field in the transistor channel, *E*, in the right-hand side of Equation (1) is expressed in *V*/*m*.

Results on both tables demonstrate the little relevance that the gate length has in coupling the radiation to the device channel. This does not mean that the gate length does not have an impact on the responsivity of the device. On the contrary, the Dyakonov-Shur theory predicted [9] that the gate length plays an important role in the detector performance in agreement with the previous measurements [21], but not in the coupling of the radiation as the length is considerably smaller than the wavelength of the radiation.

**Figure 6:**
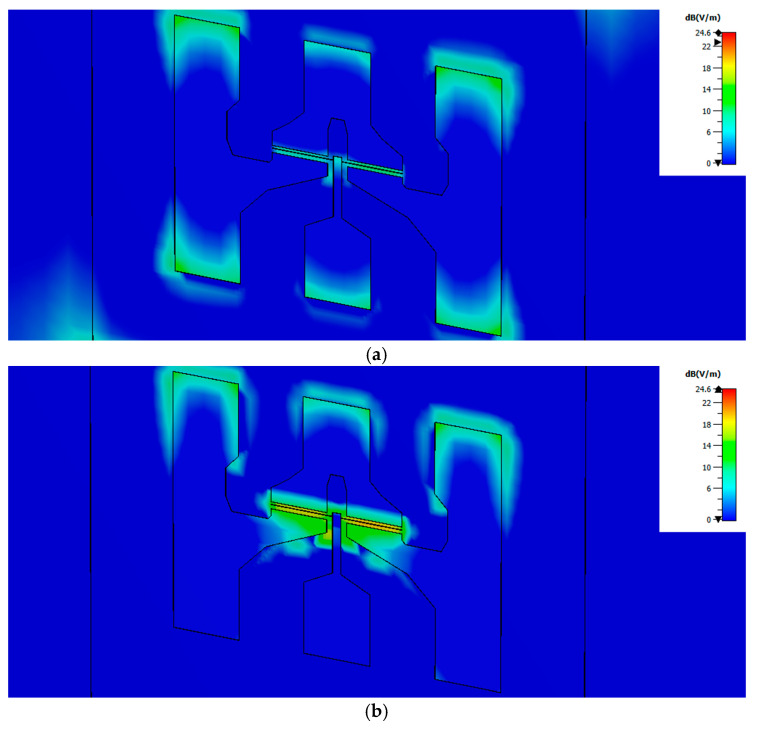
Spatial distributions of the module of the normalized electric field inside the transistor channel under illumination at 0.15 THz, (**a**), and 0.30 THz. (**b**) The maximum values of the normalized electric field inside the channel are 10.56 dB and 20.41 dB for excitation at 0.15 and 0.30 THz, respectively, as displayed in Table 1 and Table 2. Black solid lines indicate the contour of the top side transistor electrodes and are a guide to the eye.

From results in Table 1 it follows that modifying the frequency of the radiation barely changes the magnitude of the field induced in the channel when the electric field of the exciting beam is oriented along the x axis; while, in strong contrast, when the exciting electric field is oriented along the y axis the frequency of the radiation strongly modifies the *E_r_*. This last behavior is in agreement with the experimental results (Figure 4), because the measured photoresponse of the MODFET changes with the frequency of the excitation. In terms of the influence of the coupling of the incoming radiation on the photoresponse of the strained-Si MODFET the most relevant component of the incoming electric field is the one oriented along the y axis as it is added to the photovoltaic response of the device. Moreover, in the study it was systematically found that the electric field induced in the transistor by the exciting beam was essentially limited to the channel layer of the FET in agreement with Dyakonov and Shur theoretical model. Figure 6 presents the normalized electric field in the x-y plane located at the z-coordinate corresponding to the vertical position of the transistor channel, i.e., Figure 6 gives the distribution of *E_r_* inside the transistor channel for the two excitation frequencies considered. The results, just as the maxima reported on Table 1 and Table 2, shows a weaker response at 0.15 THz, i.e., a lesser effective coupling of the THz radiation into the channel, while the experimental photoresponse exhibits the opposite behavior (see Figure 4).

**Figure 7:**
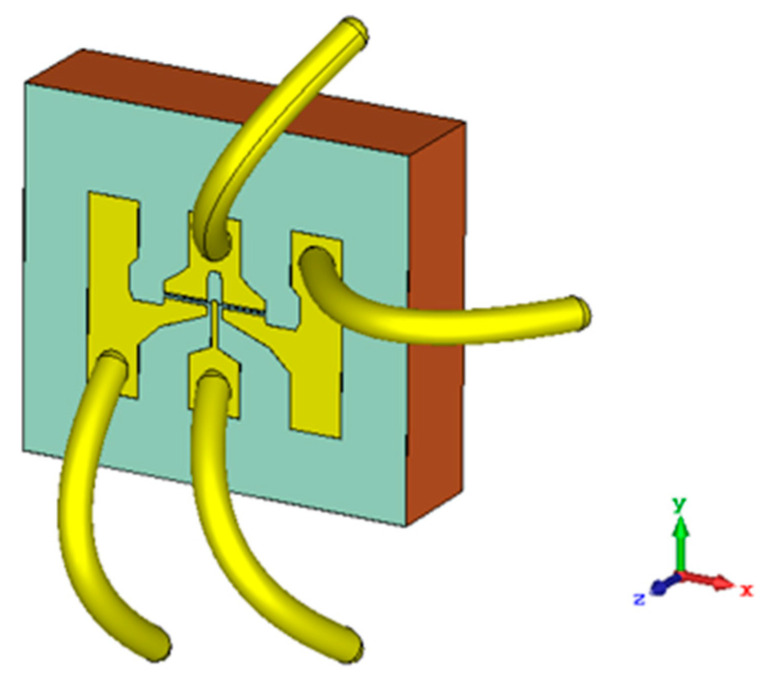
3D view of the structure used in simulations showing the bonding wires.

In the previous description of the characterization procedure it was pointed out that the strained-Si MODFET was wire-bounded on a DIP8. Since the wavelength of the radiation source is within the scale of 1 mm the coupling of the radiation through the gold wires used to contact the device must be also studied. Accordingly, the simulated structure showed on Figure 5 was modified to add four gold wires 0.25-mm long (i.e., approximately the length of a quarter wavelength antenna at 300 GHz) as shown in Figure 7. The arrangement of the four wires used in simulations (Figure 7) was the same as one of the wire-bonded device used in measurements.

Table 3 and Table 4 summarize the maximum values of the normalized internal electric field (*E_r_*) inside the channel obtained in simulations along the *x* and *y* axis, respectively.

By comparing the results in Table 3 and Table 4 with the ones in Table 1 and Table 2 it follows that the bonding wires contribute very effectively to couple the THz radiation into the channel. The contribution is especially remarkable at 0.15 THz as for the MODFET with a gate length of 0.25 μm the maximum value of *E_r_* increases from 10.56 to 35.64 dB for an exciting beam along the *y* axis; while a similar less pronounced behavior is found when the excitation is along the *x* axis. For the excitation at 0.30 THz the inclusion of the bonding wires led to a slight increment of the maximum of *E_r_* (less than 5 dB) for a beam with its electric field oriented along the y axis, while no increment was found when the exciting beam was oriented along the *x* axis.

**Figure 8:**
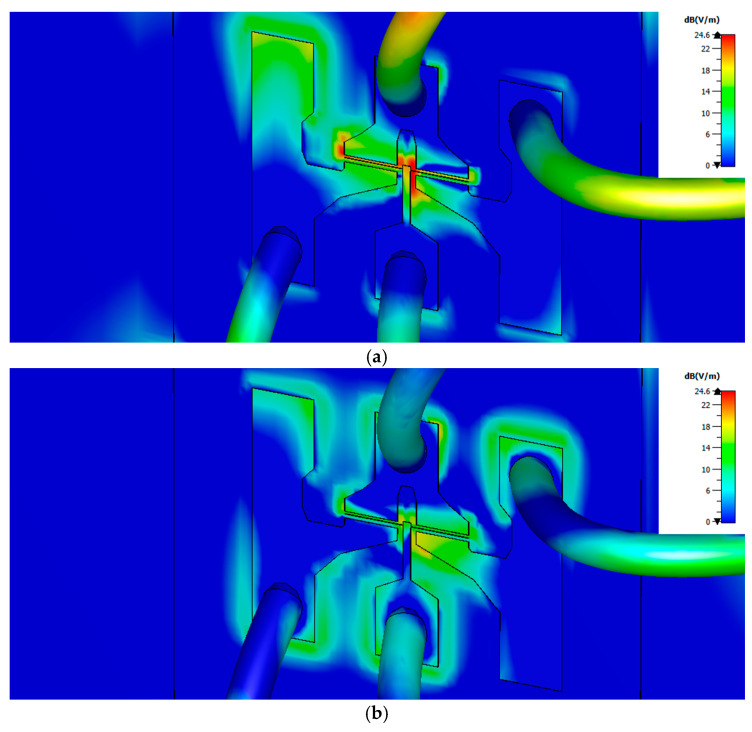
Spatial distributions of the module of the normalized electric field inside the transistor channel under illumination at 0.15 THz, (**a**), and 0.30 THz, (**b**), when the four 0.25 μm long bonding wires were included in simulations. The maximum values of the normalized electric field inside the channel are 35.64 dB and 25.49 dB for excitation at 0.15 and 0.30 THz, respectively, as displayed on Table 3 and Table 4. Black solid lines indicate the contour of the top side transistor electrodes and are a guide to the eye.

Figure 8 shows the spatial distributions of the normalized electric field inside the transistor channel for excitations at 0.15 and 0.30 THz. By comparing Figure 8 with Figure 6 an intense coupling of the incoming radiation into the channel through the bonding wires may be found, specially at 0.15 THz.

## 4. Conclusions

This paper reports on a study of the response of a T-gate strained-Si MODFETs (modulation-doped field-effect transistor) to front sub-THz excitation. The transistor is based on the Si/SiGe material system that provides excellent values of both the carrier mobility in the transistor channel and carrier confinement inside the strained Si layer where a dual channel is formed. The device was characterized using a two-tones solid-state continuous wave source at 0.15 and 0.30 THz. In agreement with the previous results, the device response in the photovoltaic mode was found to be non-resonant. The maximum of the photoresponse was clearly higher under THz illumination at 0.15 THz than at 0.3 THz.

A numerical study was conducted using three-dimensional (3D) electromagnetic simulations to delve into the coupling of THz radiation to the channel of the transistor. 3D simulations solving the Maxwell equations using a time-domain solver were performed. The exciting THz beam was modelled as a plane wave with normal incidence on the top of the transistor. 

Initially, the simulations were conducted on a purely planar structure, i.e., disregarding the bonding wires used to contact the transistor pads in experiments, using the real dimensions of both the vertical and horizontal layouts of the strained-Si MODFET. Results showed an irrelevant role of the gate length in the coupling of the radiation to the device channel. Simulations, in contradiction with measurements, pointed to a better response at 0.3 THz than under 0.15 THz excitation in terms of the normalized electric field induced by the THz beam inside the channel.

Subsequently, 0.25 mm long bonding wires were added to the model of the transistor previously used to investigate the impact of the wire-bonding used to contact the samples used in experimental measurements. Simulation results revealed the important role played by the bonding wires at the lower frequency used in the study (0.15 THz). The normalized internal electric field along the transistor channel by the 0.15 THz beam was increased in 25 dB when the bonding wires were considered and, accordingly, the overall response was found to be in agreement with the measurements. Therefore, in the lower portion of the THz spectral region, bonding wires provide an unintended improvement of the transistor response whose value is difficult to predict as it depends on the bonding wires length and their spatial arrangement. Nevertheless, at higher frequencies the detector response became relatively independent of the bonding wires. Since simulations showed that, when the effect of the wires was not taken into account, the coupling of the THz radiation to the structure was stronger at 0.3 THz than at 0.15 THz the transistor may exhibit independence from wiring along with a stronger coupling of the radiation for frequencies above 0.3 THz.

## Figures and Tables

**Table 1:** Maximum value of the normalized electric field component along x axis in dB for frequencies 0.15 and 0.30 THz.

*Lg* (μm)	0.15 THz	0.30 THz
0.50	22.0794 dB	24.5535 dB
0.25	22.0863 dB	24.5226 dB
0.15	22.0893 dB	24.5115 dB
0.10	22.0907 dB	24.5063 dB

**Table 2:** Maximum value of the normalized electric field component along y axis in dB for frequencies 0.15 and 0.30 THz.

*Lg* (μm)	0.15 THz	0.30 THz
0.50	11.2471 dB	20.7173 dB
0.25	10.5597 dB	20.4073 dB
0.15	10.4940 dB	20.2211 dB
0.10	10.3451 dB	20.1707 dB

**Table 3:** Maximum value of the normalized electric field component along x axis in dB for frequencies 0.15 and 0.30 THz considering the effect of the bonding wires.

*Lg* (μm)	0.15 THz	0.30 THz
0.50	32.9939 dB	23.8939 dB
0.25	33.0075 dB	23.9190 dB
0.15	33.0081 dB	23.9220 dB
0.10	33.0085 dB	23.9233 dB

**Table 4:** Maximum value of the normalized electric field component along y axis in dB for frequencies 0.15 and 0.30 THz considering the effect of the bonding wires.

*Lg* (μm)	0.15 THz	0.30 THz
0.50	35.7280 dB	25.5638 dB
0.25	35.6415 dB	25.4949 dB
0.15	35.6404 dB	25.4947 dB
0.10	35.6399 dB	25.4947 dB
